# Dabrafenib for cutaneous melanoma infiltrating the vitreous: regression of metastasis and occurrence of uveitis as a secondary effect

**DOI:** 10.1186/s12348-017-0135-2

**Published:** 2017-07-25

**Authors:** Alex Fonollosa, Jose Gabriel Vargas-Kelsh, Gonzaga Garay-Aramburu, Angel Saiz, Ignacio Zabalza-Estevez, Ricardo Fernandez

**Affiliations:** 1 Begiker-Ophthalmology Research Group, Ophthalmology Department, BioCruces Health Research Institute, Cruces Hospital, University of the Basque Country Plaza de Cruces s/n, 48903 Cruces-Barakaldo, Vizcaya Spain; 2 Instituto Oftalmológico Bilbao Berastegui 4, 1, 48001 Bilbao, Spain; 3 Department of Ophthalmology, Araba University Hospital José Atxotegi s/n, 1009 Vitoria-Gasteiz, Spain; 4 Department of Pathology, Galdakao-Usansolo Hospital, Barrio Labeaga s/n, 48960 Usansolo, Vizcaya Spain; 5 Department of Oncology, Clinica Zorrozaure, Ballets Olaeta Kalea 4, 48014 Bilbao, Vizcaya Spain

**Keywords:** Dabrafenib, Cutaneous melanoma, Intraocular metastatic cutaneous melanoma, Metastatic cutaneous melanoma, Uveitis

## Abstract

Intraocular metastasis of cutaneous melanoma is extremely infrequent. This typically occurs in advanced metastatic disease and has a poor survival prognosis. The most frequent reported treatment is radiotherapy. BRAF inhibitors are new, orally administered and very effective drugs used for metastatic cutaneous melanoma. Herein, we report a case of a 58-year-old patient with a recent diagnosis of cutaneous melanoma who consulted for floaters and presented vitreous opacities in both eyes. A diagnostic vitrectomy of his left eye was performed and pathologic analysis disclosed infiltrating melanoma cells in the vitreous. Treatment with dabrafenib (a type of BRAF inhibitor) achieved the regression of the intraocular metastasis in the right eye. Moreover, the patient presented a severe anterior uveitis due to dabrafenib, a well-known secondary effect of this drug.

## Introduction

Inhibitors of serine/threonine protein kinase B-Raf (so-called BRAF inhibitors) have become a hopeful therapeutic option in patients with metastatic cutaneous melanoma. There are two BRAF inhibitors: dabrafenib and vemurafenib. Both are orally administered and both have shown to provide a great benefit in terms of increased survival in these patients [1]. Uveitis is a well-known secondary effect of these types of drugs [2]. We describe a patient who presented vitreous infiltration of a cutaneous melanoma, who was effectively treated with dabrafenib. Moreover, the patient presented a severe anterior uveitis secondary to this drug.

## Case report

**Fig. 1:**
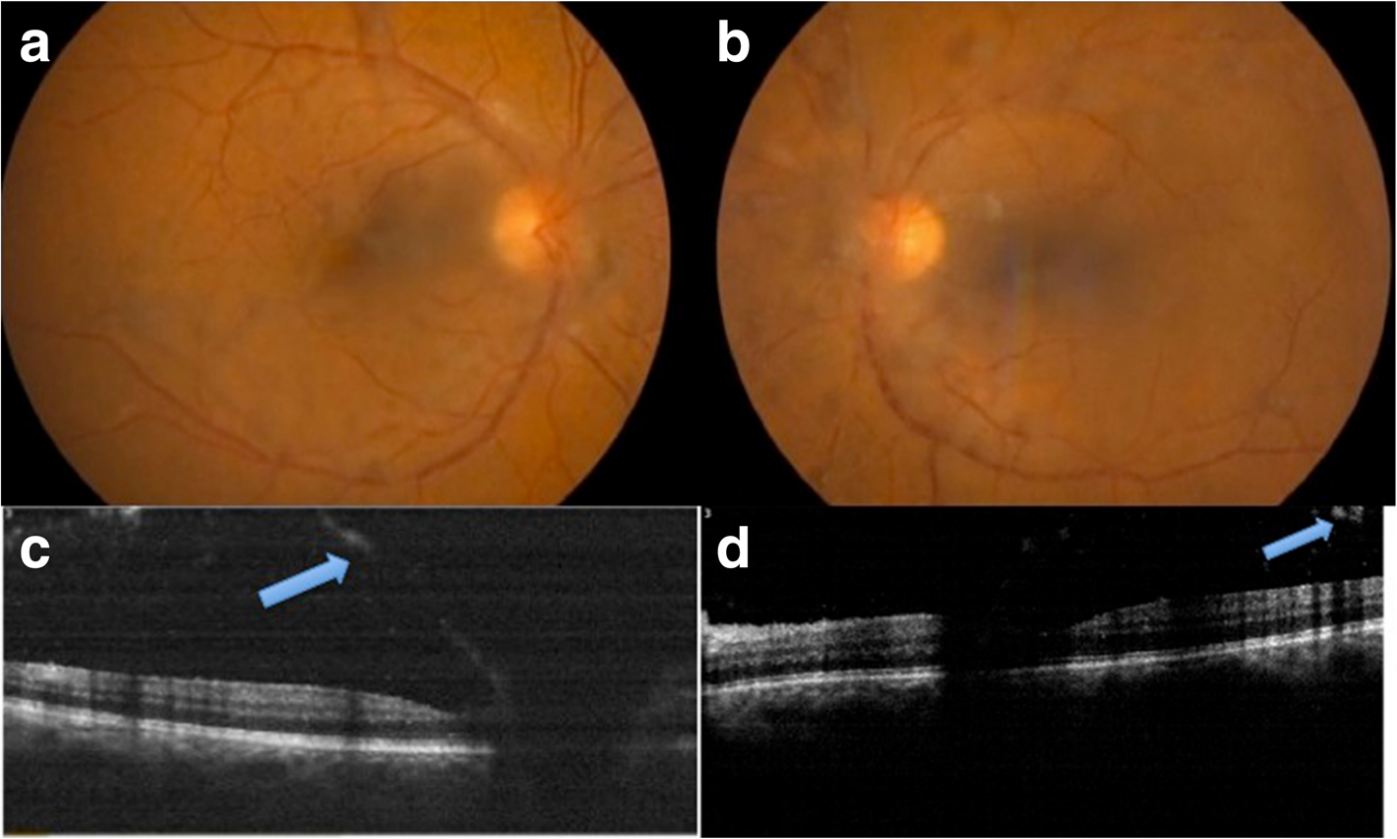
Imaging on admission. **a** and **b** Retinographies showing dense vitreous opacities; **a**: right eye, **b**: left eye. **c** and **d**: optical coherence tomography showing hyperreflective bands anterior to the retina (*blue arrows*); **c** right eye, **d**: left eye

**Fig. 2:**
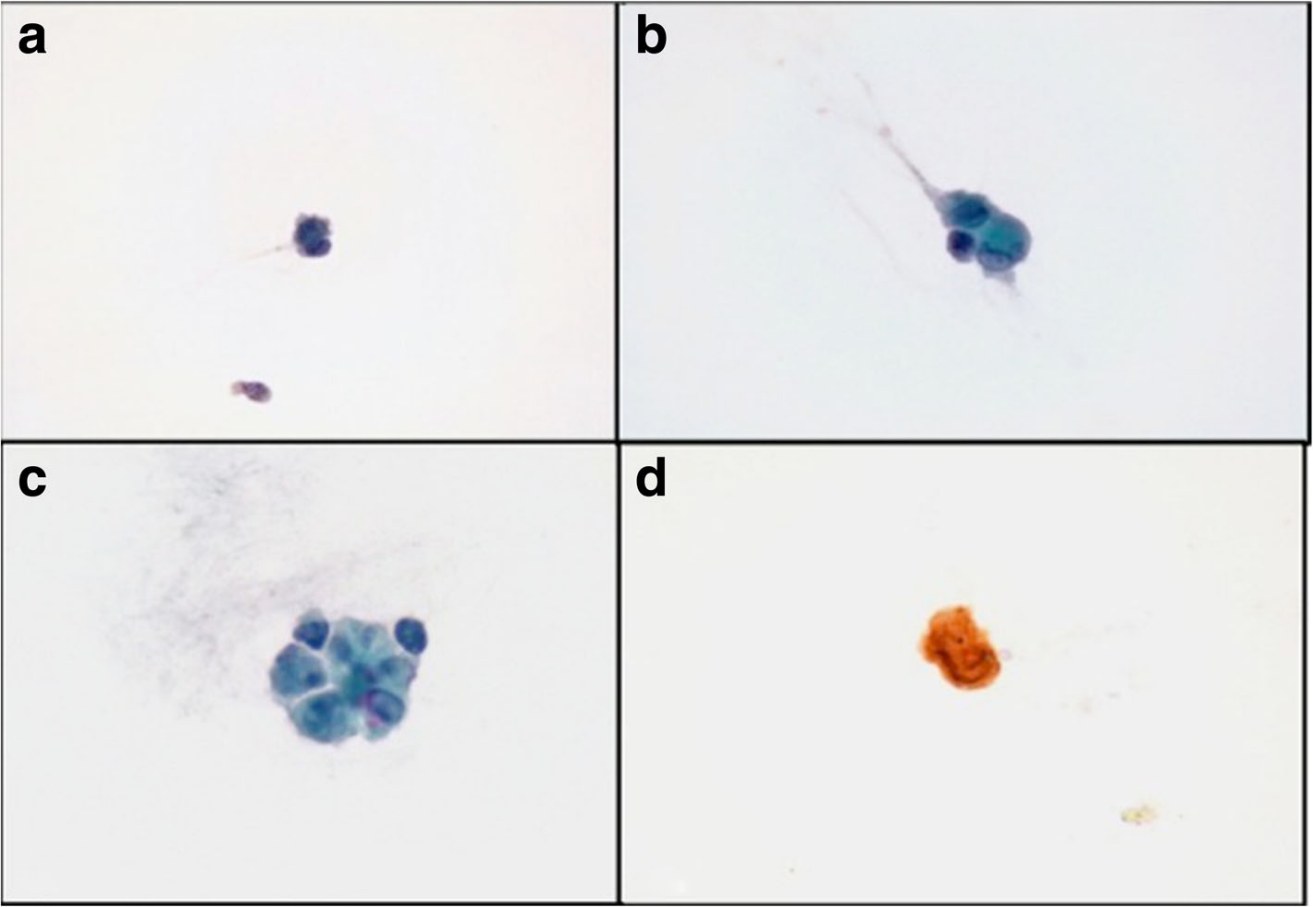
Pathologic analysis showing dissociated cells forming irregular groups with atypia (**a**–**c**), positive for HMB45 by inmunohistochemistry (**d**) consistent with a metastatic origin from a cutaneous melanoma

**Fig. 3:**
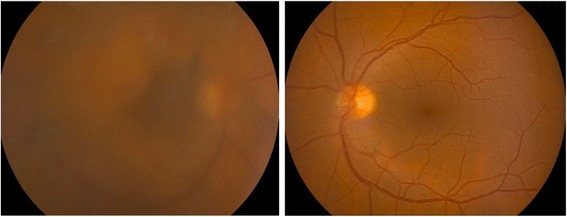
Retinographies after diagnostic vitrectomy performed in the left eye

A 58-year-old man consulted for blurry vision in both eyes of 4 weeks duration. His past medical history was relevant for cutaneous melanoma diagnosed 5 months before. It was located in the lumbar region of his back. Breslow’s depth was 1.8 mm and the sentinel node had metastasis. He received surgery for this tumour and, at the moment of consultation, he was under treatment with adjuvant interferon alpha (20 million IU administered subcutaneously three times a week). Visual acuity was 20/32 in his right eye and 20/50 in his left eye; anterior segment biomicroscopy revealed few cells and intraocular pressure was 14 mmHg in both eyes. Funduscopy revealed dense whitish vitreous opacities without signs of chorioretinal inflammation. Optical coherence tomography showed hyperreflective bands anterior to the retina corresponding to the vitreous opacities (Fig. 1). A diagnostic vitrectomy was performed in the left eye and the cytological study revealed dissociated cells forming irregular groups with atypia, positive for homatropine methylbromide 45 (HMB45) by inmunohistochemistry, consistent with a metastatic origin from a cutaneous melanoma (Figs. 2 and 3).

**Fig. 4:**
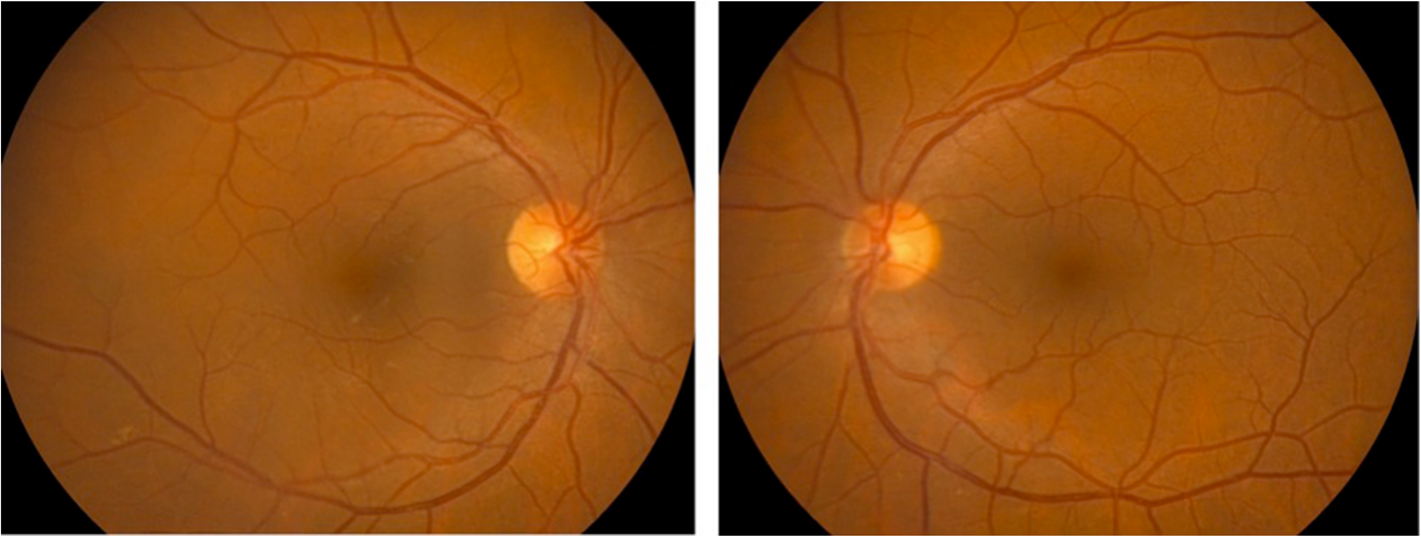
Retinographies performed after 16 weeks of dabrafenib treatment showing complete resolution of vitreous opacities in the right eye

With the diagnosis of metastatic cutaneous melanoma, and since the melanoma was positive for the V600 mutation, interferon was replaced by dabrafenib. Brain and orbital magnetic resonance imaging were performed to rule out extraocular involvement, with negative results. After 10 weeks of treatment with dabrafenib the patient consulted for blurry vision and redness in his RE. Examination revealed severe anterior uveitis and hypopyon. Topical prednisolone with tapering doses and cycloplegic drops were started, which successfully resolved the uveitis. This was attributed to dabrafenib. A new ophthalmological evaluation was performed after 16 weeks of dabrafenib treatment, revealing a complete absence of vitreous opacities in the left eye (the vitrectomised eye) as well as in the right eye, with visual acuity 20/20 in both eyes (Fig. 4). Another examination performed after 24 weeks of dabrafenib treatment again showed complete absence of cellular infiltration of the vitreous cavity in both eyes. Unfortunately, the patient was diagnosed with liver metastasis in the 32nd week of treatment and died shortly after.

## Discussion

Metastasis of cutaneous melanoma to the eye is rare and has a very poor survival prognosis. The uvea has been found to harbour the majority of intraocular cutaneous melanoma, with choroidal involvement occurring in more than half of the cases [3]. Isolated vitreous metastases are exceedingly rare with only a few cases reported in the literature [4]. Different treatments have been reported for its management, with radiotherapy the most frequent. Other options include enucleation or evisceration, vitrectomy, subconjunctival chemotherapy, laser photocoagulation and cryotherapy [3]. We used one of the drugs approved for the treatment of metastatic cutaneous melanoma, dabrafenib. To our knowledge there are no published reports on the use of dabrafenib for cutaneous melanoma with intraocular metastasis. We postulate that dabrafenib penetrates the blood-retinal barrier well since it achieved a complete resolution of vitreous infiltration in our case. Interestingly, a good penetration of the blood-brain barrier by this drug has been suggested due to its high clinical activity for central nervous system metastasis of cutaneous melanoma in a clinical trial [5]. Uveitis is a well-known secondary effect of BRAF inhibitors. We recently described a series of patients with uveitis secondary to vemurafenib that resolved with local steroids and temporary withdrawal of vemurafenib [6]. Our case developed a severe anterior uveitis which also responded to topical steroids. Typically uveitis secondary to BRAF inhibitors is bilateral and our case was unilateral. One potential mechanism for the origin of the uveitis after BRAF inhibitor use is a direct action on intraocular metastatic cells [2]. This may explain why our patient presented with unilateral uveitis involving only the infiltrated non-vitrectomised eye.

In conclusion, the known efficacy of dabrafenib for treating metastatic disease of cutaneous melanoma replicated when the metastasis involved the vitreous. This might be a safe and effective alternative for managing this infrequent type of metastasis.

## Abbreviations

BRAF: Serine/threonine protein kinase B-Raf
HMB45: Homatropine methylbromide 45
